# Compensatory quadrant-hyperhidrosis after contralateral intrathoracic surgery: a case report

**DOI:** 10.1186/1752-1947-7-24

**Published:** 2013-01-18

**Authors:** Stefan Brodoehl, Otto Wilhelm Witte, Albrecht Guenther

**Affiliations:** 1 Hans Berger Department of Neurology, Jena University Hospital, Erlanger Allee 101, D 07747, Jena, Germany

**Keywords:** Botulinum toxin, Intrathoracic surgery, Unilateral hyperhidrosis

## Abstract

**Introduction:**

Unilateral hyperhidrosis can be a neurological manifestation of irritations of the central or peripheral nervous system.

**Case presentation:**

We present the case of a 67-year-old German man who had hyperhidrosis of his right upper body quadrant (including face, arm, and chest) following intrathoracic surgery of a left-sided pleural lipoma.

**Conclusion:**

An isolated unilateral hyperhidrosis might occur after intrathoracic surgery. Besides anticholinergic drugs the use of botulinum toxin should be considered.

## Introduction

Anhidrosis and hyperhidrosis can be neurological manifestations of irritations of the central or peripheral nervous system [1,2]. There are described causes such as injuries, tumors, infarcts or hemorrhages of the brain or the medulla.

## Case presentation

A 67-year-old German man admitted to our botulinum toxin clinic complained about extensive sweating of his right upper body including his right facial area, right arm, and chest, whereas the left side of his body appeared to be ‘summer-dry’. At the time of his presentation at our clinic the patient reported a subjective reduction of quality of life. The massive hyperhidrosis made him feel uncomfortable in the company of strangers and therefore kept him from taking part in social activities.

Symptoms started some weeks after tumor extirpation of a left-sided pleural lipoma via an anterior-posterior thoracotomy at the 5th intercostal space in conjunction with a partial pleurectomy. After being released from hospital, he experienced contralateral hyperhidrosis which started in the face spreading downwards.

Clinical neurological examination together with ophthalmological tests yielded a normal status appropriate to the patient’s age. Sympathetic skin nerve action potentials [3] were recorded in both arms and legs and revealed a significantly decreased sympathetic activity in the left arm. Pharmacological pupil function test (cocaine and phenylephrine eye drops) showed no discrepancy on the left side. In particular, Horner’s syndrome or hypesthesia were not found. In addition, scintigraphy showed no proof of a reduced function of salivary glands. After physical activity, a starch-iodine preparation was performed, revealing significant hyperhidrosis of the right upper body together with a relevant temperature difference measured on the chest (right 30.5°C, left 33.7°C). Lung auscultation, before and after physical exertion, yielded right-sided bronchial spasm post-exercise.

Treatment was started with bornaprine: a central anticholinergic drug. A starting dose of two mg per day increasing to six mg did not lead to a relevant clinical improvement but produced intolerable side effects.

Due to a considerable subjective impairment, we decided on a test injection of botulinum toxin type A. A total of 20 units at three injection points on the right side of the patient’s forehead and a total of 40 units at six injection points on his right upper body were administered. Some improvement was achieved and therapy was repeated in a three-month interval.

## Discussion

The localized unilateral hyperhidrosis in the present case developed after intrathoracic surgery. Therefore, we hypothesize that the quadrant-hyperhidrosis of the right upper body is a (hyper-) compensation of a disturbed sympathetic innervation of the contralateral left side due to a surgical lesion of the cervical sympathetic chain. The lesion is assumed to be situated in the endothoracic fascia, where the cervical sympathetic fibers are closely related to the apical pleura [4]. Presumably caused by a disturbed negative feedback of afferent sympathetic signals, the sweating center in the hypothalamus generates an increased positive feedback signal that induces severe sweating on the body side contralateral to the sympathetic lesion [5].

Table 1 provides an overview of known cases of localized unilateral hyperhidrosis in the literature and (if included) briefly describes applied therapies and their effectiveness. Taken together most successful therapy strategies included the application of botulinum toxin type A. Besides sympathectomy treatment with antidepressants or local therapy with aluminum hexachloride have proven effective. Although not effective in the present case, the use of oral anticholinergic drugs is a good therapeutic alternative especially because Wolosker *et al*. [6] have recently shown the potential of oxybutynin in treating localized hyperhidrosis.

**Table 1:** Cases of localized unilateral hyperhidrosis in the literature excluding isolated facial hyperhidrosis

**Cause of hyperhidrosis**	**Reference**	**Cases**	**Therapy**
*Idiopathic unilateral hyperhidrosis*			
	Cunliffe *et al*. [7]	3	local anticholinergic*
	van de Kerkhof *et al*. [8]	1	no
	Dworin and Sober [9]	1	local Al_2_Cl_6_ *
	Fernández and Armijo [10]	1	local Al_2_Cl_6_ *
	Querol Nasarre *et al*. [11]	1	no
	Köse and Baloglu [12]	1	no
	Boyvat *et al*. [13]	1	antidepressant
	Kreyden *et al*. [14]	1	botulinum toxin A *
	Kocyigit *et al*. [15]	1	no
*Intrathoracic neoplasia*			
	Middleton [16]	1	no
	Walsh *et al*. [17]	2	no
	McCoy [18]	1	no
	Wang *et al*. [19]	2	no
	McEvoy *et al*. [20]	1	no
	Lindsay *et al*. [4]	2	no
	Umeki *et al*. [21]	1	no
	Lambert *et al*. [22]	1	local radiotherapy *
	Yamauchi *et al*. [23]	1	no
	Slabbynck *et al*. [24]	1	no
	Lee and Greenstone [25]	1	no
	Nishimura *et al*. [26]	1	local radiotherapy *
	Sribnick and Boulis [27]	1	sympathectomy *
*Stroke* – *brainstem infarction*			
	Mon and Mizotani [28]	1	no
	Korpelainen *et al*. [29]	16	no
	Rey *et al*. [30]	1	no
	Rousseaux *et al*. [31]	5	no
	Sato and Nitta [32]	1	no
	Pellecchia *et al*. [33]	1	no
*Stroke* – *cerebral infarction*			
	Labar *et al*. [34]	2	no
	Sakashita *et al*. [35]	1	no
	Kim *et al*. [36]	5	no
	Bassetti and Staikov [37]	1	no
	Smith [38]	1	no
	Faruqi *et al*. [39]	1	no
*Postinterventional complications*			
	Belin and Polo [40]	1	botulinum toxin A *
	Aşik *et al*. [41]	1	local radiotherapy *
*Spinal cord pathology*			
	Pool [42]	1	hemilaminectomy *
	Baskan *et al*. [43]	1	botulinum toxin A *
	Kilinçer *et al*. [44]	1	no
	Gorman [45]	1	improved bedding *
*Sweat glands pathology*			
	Kopera and Soyer [46]	1	appetite depressant *
	Ruiz de Erenchun *et al*. [47]	1	antidepressant *
	Parslew and Lewis-Jones [48]	1	no
*Others* (*Buerger's disease*)			
	Baker [49]	1	no

(*) indicates successful therapy, (Al_2_Cl_6_) aluminum hexachloride.

Another relevant clinical aspect might be a bronchial spasm of the right lung in the course of physical exertion due to an overwhelming activity of the left-sided parasympathetic nervous system. In our case, the patient had no medical history of chronic lung disease; although an obstructive lung disease cannot be excluded beyond doubt. This additional feature might be an interesting physiological aspect that has – to the best of our knowledge – never been reported before.

## Conclusion

An isolated unilateral hyperhidrosis is a rare complication after intrathoracic surgery that potentially reduces the quality of life. As therapeutic options anticholinergic drugs or the use of botulinum toxin could be considered.

## Consent

Written informed consent was obtained from the patient for publication of this manuscript and accompanying images. A copy of the written consent is available for review by the Editor-in-Chief of this journal.

## Competing interests

The authors declare that they have no competing interests.

## Authors’ contribution

SB and AG performed the clinical examination, analyzed and interpreted the diagnostic findings, and applied the pharmacological therapy. Conception and discussion was performed by all authors. The main writing was done by SB. All authors read and approved the final manuscript.
